# Severe SARS-CoV-2 Breakthrough Reinfection With Delta Variant After Recovery From Breakthrough Infection by Alpha Variant in a Fully Vaccinated Health Worker

**DOI:** 10.3389/fmed.2021.737007

**Published:** 2021-08-20

**Authors:** Jayanthi Shastri, Swapneil Parikh, Veena Aggarwal, Sachee Agrawal, Nirjhar Chatterjee, Rajit Shah, Priti Devi, Priyanka Mehta, Rajesh Pandey

**Affiliations:** ^1^Kasturba Hospital for Infectious Disease, Mumbai, India; ^2^IJCP Group & Heart Care Foundation of India, New Delhi, India; ^3^INtegrative GENomics of HOst-PathogEn Laboratory, CSIR-Institute of Genomics and Integrative Biology, New Delhi, India; ^4^Academy of Scientific and Innovative Research, Ghaziabad, India

**Keywords:** SARS-CoV-2, COVID-19, reinfection, breakthrough, whole genome sequencing, breakthrough reinfection

## Abstract

**Background:** Post infection immunity and post vaccination immunity both confer protection against COVID-19. However, there have been many whole genome sequencing proven reinfections and breakthrough infections. Both are most often mild and caused by Variants of Concern (VOC).

**Methods:** The patient in our study underwent serial COVID-19 RT-PCR, blood tests for serology, acute phase reactants, and chest imaging as part of clinical care. We interviewed the patient for clinical history and retrieved reports and case papers. We retrieved stored RT-PCR positive samples for whole genome sequencing (WGS) of SARS-CoV-2 from the patient's breakthrough infections and the presumed index case.

**Findings:** The patient had three RT-PCR confirmed SARS-CoV-2 infections. Two breakthrough infections occurred in quick succession with the first over 3 weeks after complete vaccination with COVISHIELD and despite post-vaccination seroconversion. The first breakthrough infection was due to the Alpha variant and the second due to the Delta variant. The Delta variant infection resulted in hypoxia, hospitalization, and illness lasting seven weeks. Serial serology, acute phase reactants, and chest imaging supported WGS in establishing distinct episodes of infection. WGS established a fully vaccinated family member as the index case.

**Interpretation:** The patient had an Alpha variant breakthrough infection despite past infection, complete vaccination, and seroconversion. Despite boosting after this infection, the patient subsequently had a severe Delta variant breakthrough infection. This was also a WGS proven reinfection and, therefore, a case of breakthrough reinfection. The patient acquired the infection from a fully vaccinated family member.

## Introduction

A year and a half into the Coronavirus Disease 2019 (COVID-19) pandemic, we are at the heart-wrenching milestone of four million recorded COVID-19 deaths. Estimates of the actual number of deaths based on excess deaths are staggeringly higher at over ten million (1). The true number of infections is estimated to be five to twenty times higher than the number of confirmed cases (2) and runs into billions. Fortunately, we have several effective vaccines with which we can potentially contain the COVID-19 pandemic. Unfortunately, our vaccination efforts have to contend with rapidly spreading Severe Acute Respiratory Syndrome Coronavirus 2 (SARS-CoV-2) variants, and that is a monumental challenge.

In December of 2020, the Alpha variant was detected in the UK (3) and designated the first variant of concern (VOC). Since then, it has been a race between the speed of COVID-19 vaccine distribution and the emergence and spread of SARS-CoV-2 variants.

While the number of vaccinated individuals has crossed one billion worldwide, new variants of concern have emerged that are more concerning than their predecessors, with the Delta variant being the most concerning variant so far. The variants are concerning because of increased transmissibility, increased disease severity, and immune escape resulting in the risk of reinfections in convalescent individuals or breakthrough infections in vaccinated individuals (4).

Between February and June of 2021, India experienced a second wave of COVID-19, partially attributable to VOCs (5). Both the Alpha and Delta variants have been identified in India, and have contributed to the second wave in India (5). With the Delta variant poised to become the dominant lineage worldwide, the combination of increased transmissibility and immune escape is perilous.

Breakthrough infections are tempered by vaccines and are generally mild (5). Vaccines also reduce the risk of onward transmission (6), but it is unclear whether this holds true for the Delta variant and all vaccines. Reinfections are thought to be relatively rare, but difficulty in retrieving paired samples from different episodes for whole genome sequencing (WGS) makes it challenging to establish reinfection. In the context of VOCs, reinfections are possible and likely more common than we think. Even when reinfections are WGS proven, serial serology, inflammatory markers, and radiological imaging are usually unavailable. This limits our understanding of these rare but immunologically significant episodes.

Individuals with immunity from natural infection and vaccination are said to have hybrid immunity. The combination of post-infection immunity and post-vaccination immunity results in antibody responses that are 25 to 100 times higher, improved memory B cell and CD4+ T cell responses, and better cross-protection against variants (7). SARS-CoV-2 infection in an individual with such hybrid immunity ought to be very rare, a severe infection even rarer still. However, proving that a breakthrough infection was also a reinfection is subject to difficulties in identifying such rare cases and retrieving samples. Therefore such cases are hard to prove.

The patient in our study had two WGS proven breakthrough infections with Alpha and Delta variants, with the second breakthrough infection resulting in hospitalization. The patient had serial serology, blood investigations, inflammatory markers, and radiological imaging giving us a unique opportunity to study this breakthrough reinfection.

## Materials and Methods

### Study Participant and Case Details Including Clinical, Investigation, and Radiological Data

The Patient sought clinical care for COVID-19 in Delhi, India. All the treatment and most of the investigations were ordered by the patient's treating doctors. Some investigations were self-initiated by the patient. The patient contacted Kasturba Hospital for Infectious Diseases for whole genome sequencing and to get a better understanding of her case. Clinical history was recorded directly from the patient during telephonic interviews. We retrieved case records, inpatient papers, reports of all investigations, and radiological images. Other than WGS on old samples, no fresh investigations were conducted for this study. The patient in our study lived with and had close contact with a confirmed COVID-19 case. The presumed index case was a fully vaccinated family member. A stored nasopharyngeal plus oropharyngeal sample (NP + OP) from the presumed index case was also retrieved for whole genome sequencing.

### RT-PCR and COVID-19 Serology

All RT-PCR tests were conducted on NP + OP swabs transported in viral transport media (VTM). The positive RT-PCR in August 2020 was performed using TRUPCR kit on Quant Studio 5 by Thermo Fisher. All positive RT-PCR samples during the first and second breakthrough infections were conducted at the same laboratory using TRUPCR kit on CFX 96 dx-Bio-Rad, allowing for a comparison of Ct values in these two episodes. The gene targets for TRUPCR kit are E gene, RdRp gene and N gene. Details on the methodology of the RT-PCR tests are presented in Supplementary Table 1.

Serological testing was conducted using chemiluminescence (CLIA) but with different kits and platforms, including Abbott anti-N IgG, Roche anti-N IgG, Abbott anti-S1 RBD IgG, Liaison Anti-S IgG, and Beckman Coulter anti-RBD IgG. The methodology for each test in mentioned in Table 1.

**Table 1:** Clinical features, RT-PCR, Ct values, and Serology.

**Event**	**RT-PCR date and result**	**Ct values**		**Clinical features, duration and severity**	**Serology**
		**E**	**RdRp + N**		
First infection episode	16/08/2020 Positive	28	28	Asymptomatic	07/09/20 Negative (Abbott anti-N IgG)
	19/08/2020 Negative	-	-		09/10/20 Negative (Roche Anti-N IgG)
					02/11/20 Negative (Abbott anti-N IgG)
COVISHIELD dose one 01/02/2021	-	-	-	-	04/02/21 Non-reactive (Beckman Coulter anti-RBD IgG)
					18/02/21 Positive 47.30 AU/ml (Liaison Anti-S IgG)
COVISHIELD dose two 15/03/2021	-	-	-	-	21/03/2021 Reactive, Index 5.84 (Beckman Coulter anti-RBD IgG)
					07/04/21 Reactive, Index 2.85 (Beckman Coulter anti-RBD IgG)
Second infection episode	12/04/2021 Positive	34.52	33.27	Abdominal pain, fever, myalgia and fatigue. Illness duration 11 days. Moderate COVID-19.	24/04/21 Positive 692.40 AU/ml (Abbott Anti-RBD IgG)
	14/04/2021 Negative	-	-		
	24/04/2021 Negative	-	-		
Third infection episode	03/05/2020 Positive	20.41	20.25	Body ache, fatigue, headache, cough, breathlessness, fever, rhinorrhea, vomitting. 6 weeks. Severe COVID-19 requiring hospitalization and supplemental oxygen.	31/05/21 Positive >40,000 AU/ml (Abbott Anti-RBD IgG)
	04/05/2021 Positive	20.09	18.20		
	08/05/2021 Positive	21.38	22.79		
	15/05/2021 Negative	-	-		
	18/05/2021 Negative	-	-		

*S, Spike; RBD, Receptor Binding Domain; N, Nucleocapsid; IgG, Immunoglobulin G*.

### RNA Isolation

Viral RNA from the VTM solution was extracted using a commercially available RNA extraction kit (QIAmp viral mini kit, Qiagen, Cat. No. 52906). 200 μl of VTM solution was processed for lysing and viral enrichment following the kit protocol. After washing with wash buffers, viral RNA was eluted in RNase-free water.

### Whole Genome Sequencing (WGS) and Analysis

WGS of SARS-CoV-2 was performed using Oxford Nanopore Technology (ONT). In brief, cDNA was prepared using the SuperScript IV first strand synthesis system (cat no.18091050), followed by RNA strand degradation using RNase H, and the second strand cDNA synthesis using DNA Polymerase I, Large (Klenow) Fragment (cat no.M0210L). 100 ng of double-stranded cDNA was used for PCR using ARTIC nCov-2019 V3 panel and Q5® Hot Start High-Fidelity 2X Master Mix (cat no. M0494L). The sequencing library was prepared as per ONT library preparation protocol (PTC_9096_v109revE_06Feb2020). In short, unique barcodes were ligated to samples using Blunt/TA Ligase Master Mix (cat no. M0367L), after end repair of the amplicons using NEBNext Ultra II End Prep Enzyme Mix (cat no. E7546L). The sequencing adapter was ligated using NEBNext® Quick Ligation Module and the final library was sequenced on the MinION Mk1C. RNA quantification and quality control was performed using Nanodrop and taking 260/280 ratio into account. With patient samples, even sometimes when the 260/280 ratio is below 1.8, the sample is taken forward for sequencing.

The ARTIC protocol was used for data analysis wherein the raw fast five files were base called and demultiplexed using Guppy. Read filtering was performed to retain read lengths of 400–700 and remove chimeric reads. MinION was used for generating consensus FASTA and detecting variants (VCF). Using 97 random COVID-19 Indian sequences from GISAID for May 2021 and the three study samples, phylogenetic analysis was performed. All the sequences were aligned to NC_045512 reference genome using MAFFT v7.475 (8). The aligned sequences were trimmed to remove gaps, and a phylogenetic tree was generated using the default model of the IQ-TREE v2.0.3 (9). The tree was visualized using FigTree v1.4.4 (10). The assembled SARS-CoV-2 genomes were assigned clade using Nextstrain (11). A lollipop plot was generated in RStudio using g3viz, rtracklayer, and trackViewer packages, followed by data visualization using the ggplot2 package. All the figures were updated using Inkscape software (https://inkscape.org).

## Results

### Clinical Details and Investigations

**Figure 1:**
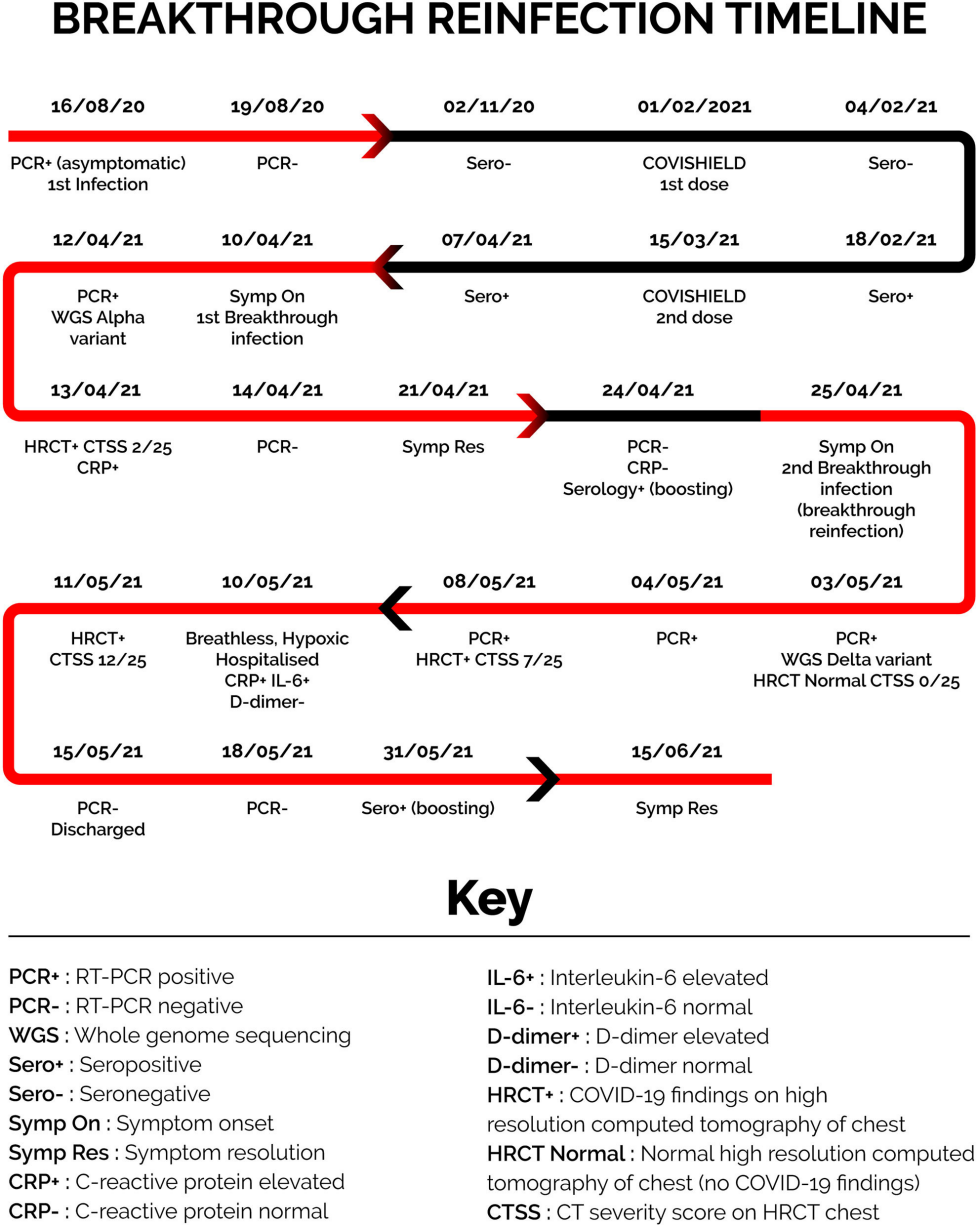
Breakthrough reinfection timeline.

The patient was a 61-year-old female health care worker in Delhi, India. She had a medical history of prediabetes for 6 months, hypertension for 2 years, and bronchial asthma since childhood. She did not have any history of immune-compromising conditions. A summary of the case is presented as a timeline in Figure 1 and of investigations in Tables 1, 2.

**Table 2:** Investigations (CRP, IL-6, D-dimer, HRCT Chest).

**Date**	**Infection episode**	**CRP (ref range <5 mg/dL)**	**IL-6 (ref range <7 pg/mL) &** **D-Dimer (ref range <0.50 mg)**	**HRCT Chest**
12/04/21	First breakthrough infection	9.0		
13/04/21		100.15		COVID pneumonia CTSS 2/25
20/04/21		33.45		
24/04/21		1.49		
27/04/21	Second breakthrough infection	4.51		
30/04/21		6.64		
03/05/21		10.88		Normal, CTSS 0/25
07/05/21			IL-6 14.29	
08/05/21		17.16		COVID pneumonia CTSS 7/25
10/05/21		251	IL-6 378, D-dimer 0.23	
11/05/21				COVID pneumonia CTSS 12/25
12/05/21		175	IL-6 <1.50	
14/05/21		46.97		
15/05/21		22.03	IL-6 <1.50, D-dimer 267.5	
16/05/21		9.74		
19/05/21		3.93		
31/05/21		2.96		

*CRP, C reactive protein; IL-6, Interleukin-6; HRCT, High-resolution computed tomography; CTSS, Computed tomography severity score*.

The first episode of SARS-CoV-2 infection was in August 2020. The patient underwent a pre-travel COVID-19 RT-PCR test on 16th August 2020, which was positive for SARS-CoV-2 (Ct values for all positive tests are presented in Table 1). A repeat test on 19th August 2020 was negative. She was entirely asymptomatic during this episode and self-isolated, and received care at home. Treatment included Tab. Ivermectin and Cap. Doxycycline. Serological testing was performed several times after this episode and before vaccination and the patient was seronegative (details are presented in Table 1).

On 1st February 2021, she received the first dose of COVISHIELD (Oxford-Astra Zeneca COVID-19 vaccine). On 18th February she was seropositive. On 15th March 2021, she received her second dose of COVISHIELD and was seropositive 6 days later on March 21st. On April 7th, 3 days before the onset of symptoms in the first breakthrough episode, serology was repeated and was positive but with a reduced index (details are presented in Table 1).

The second episode of SARS-CoV-2 infection, which was the first breakthrough infection, was in April 2021. On April 10th, the patient developed acute abdominal pain, fever, myalgia, and fatigue. The pain was in the epigastric region, acute in onset, dull aching in character, and localized. The pain was associated with tenderness but not associated with nausea, vomiting, and change in bowel or bladder habits. The pain was initially mild in intensity but progressed over the next 2 days to become severe. It resolved completely in another 3 days after starting treatment as described below. On April 10th, a few hours after the onset of abdominal pain, the patient had a single fever spike of 101°F. Fever resolved with paracetamol without any further spikes. Two days after symptom onset, the patient developed severe body ache and extreme exhaustion that persisted for 10 days, at which point all symptoms resolved, and the patient felt completely well. She did not experience sore throat, cough, breathlessness, nasal congestion, rhinorrhoea, change in smell or taste at any point in the illness. Pulse oximetry was performed daily during this episode, and her oxygen saturation was normal throughout, with values between 97 and 99%.

Two days after symptom onset (April 12th), she underwent RT-PCR testing, which was positive for SARS-CoV-2 RNA. This sample was retrieved for whole genome sequencing as detailed later. RT-PCR was negative for SARS-CoV-2 RNA 4 days (April 14th) and 14 days (April 24th) after symptom onset. Ct values are presented in Table 1.

During this episode, she had serial blood tests, including C-reactive protein (CRP). CRP peaked on April 13th, and reduced progressively to normal on April 21st. Details for CRP are presented in Table 2.

Three days after symptoms onset (April 13th), she underwent chest high resolution computed tomography (HRCT), which revealed subtle ill-defined ground glass opacification in the posterior and lateral basal segments of the bilateral lower lobes suggestive of viral pneumonitis, with a CT severity score of 2/25.

She self-isolated and received treatment at home. Treatment included T. Azithromycin, T. Ivermectin, T. Rivaroxaban, and T. Prednisolone (40 mg daily for 10 days). By April 21st, her symptoms had resolved completely, and RT-PCR on April 24th was negative for SARS-CoV-2 RNA.

The third episode of SARS-CoV-2 infection, which was the second breakthrough infection, was in late April 2021, and continued into May with some symptoms persisting until mid-June. On April 25th, the patient developed body ache, fatigue, and headache, which was later accompanied by cough, fever, rhinorrhea, vomiting, and breathlessness. Initially, the patient experienced body ache, fatigue, and headache and thought it was related to the prior infection. However, over the next 2 days, she developed a cough, initially dry but which over the next 2 days became productive with expectoration of small volumes of yellowish sputum. The cough worsened with bouts of coughing coming more frequently and lasting longer. Over the next few days, it progressed so that the patient would start coughing continuously on walking just a few steps or with the slightest exertion. Cough was not associated with hemoptysis or chest pain. Seven days into this episode (May 2nd), the patient developed a fever, which was continuous, and was associated with chills and rigor. Fever spikes continued for 2 weeks.

One week after the onset of fever (May 10th), the patient started to feel breathless at rest, and for the first time, pulse oximetry revealed hypoxia with oxygen saturation of 93%. She was hospitalized on the same day and started on supplementary low flow oxygen therapy. She remained in hospital for 5 days, and her oxygen saturation fluctuated between 93 and 97%. After 5 days of hospital care, she was discharged; fever and breathlessness had resolved, and oxygen saturation was normal on room air. Residual fatigue, body ache, and cough persisted for a month after discharge.

A day after the onset of fever (May 3rd), the patient underwent RT-PCR for the first time in the third episode, and it was positive for SARS-CoV-2. This sample was retrieved for whole genome sequencing as detailed later. Serial RT-PCR tests were positive with a reduction in Ct values. RT-PCR was negative on discharge from the hospital on May 15th. Details of RT-PCR tests and Ct values are presented in Table 1.

The patient underwent serial blood investigations in the third episode. CRP, which was normal on April 24th, increased progressively over the episode. CRP was elevated on April 30th and peaked on May 10th. Thereafter as the patient clinically improved, the CRP reduced progressively. Interleukin-6 progressively increased to a peak on May 10th. D-dimer was normal on the day of hospitalization (May 10th), indicating that the breathlessness and hypoxia were not due to a pulmonary embolus. D-dimer increased to a peak on May 15th. Details for CRP, IL-6, and D-dimer are presented in Table 2.

Serial chest HRCT was performed during this episode. An HRCT chest on May 3rd reported no abnormalities with a CTSS of 0/25. HRCT chest on May 8th revealed COVID-19 pneumonia with a CTSS of 7/25. HRCT chest on May 11th revealed progression of pulmonary involvement with multiple non-segmental areas of ground glassing and associated interlobular septal thickening involving bilateral lungs with intervening areas of consolidation with an increased CTSS of 12/25.

Treatment in the hospital included low flow oxygen therapy, Remdesivir, Dexamethasone, Enoxaparin, Paracetamol, and Cefepime. In addition, the patient, required insulin to manage hyperglycemia secondary to steroids. On discharge, the patient was prescribed Rivaroxaban, Novorapid, and a tapering dose of Prednisolone.

The patient made a complete recovery, and subsequent serological testing demonstrated boosting of humoral immunity (details have been presented in Table 1).

### Close Contact With a Confirmed Case of COVID-19

The patient lived with a fully vaccinated family member who developed symptoms of COVID-19 3 days before the patient developed symptoms in the third episode. The patient cared for and had close contact with this family member, who was presumed to be the index case. The presumed index case was fully vaccinated and had taken the second dose 2 months prior to symptom onset. An RT-PCR positive sample from 27th April 2021 was retrieved for whole genome sequencing. The patient was self-isolating at home in a separate room due to the previous infection, and the only potential exposure to infection was with the fully vaccinated unwell family member during caregiving activities. There was no other potential exposure to infection.

### Genomic Analysis Reveals Different Variants of Concern in Each Breakthrough Infection and Establishes the Index Case

**Figure 2:**
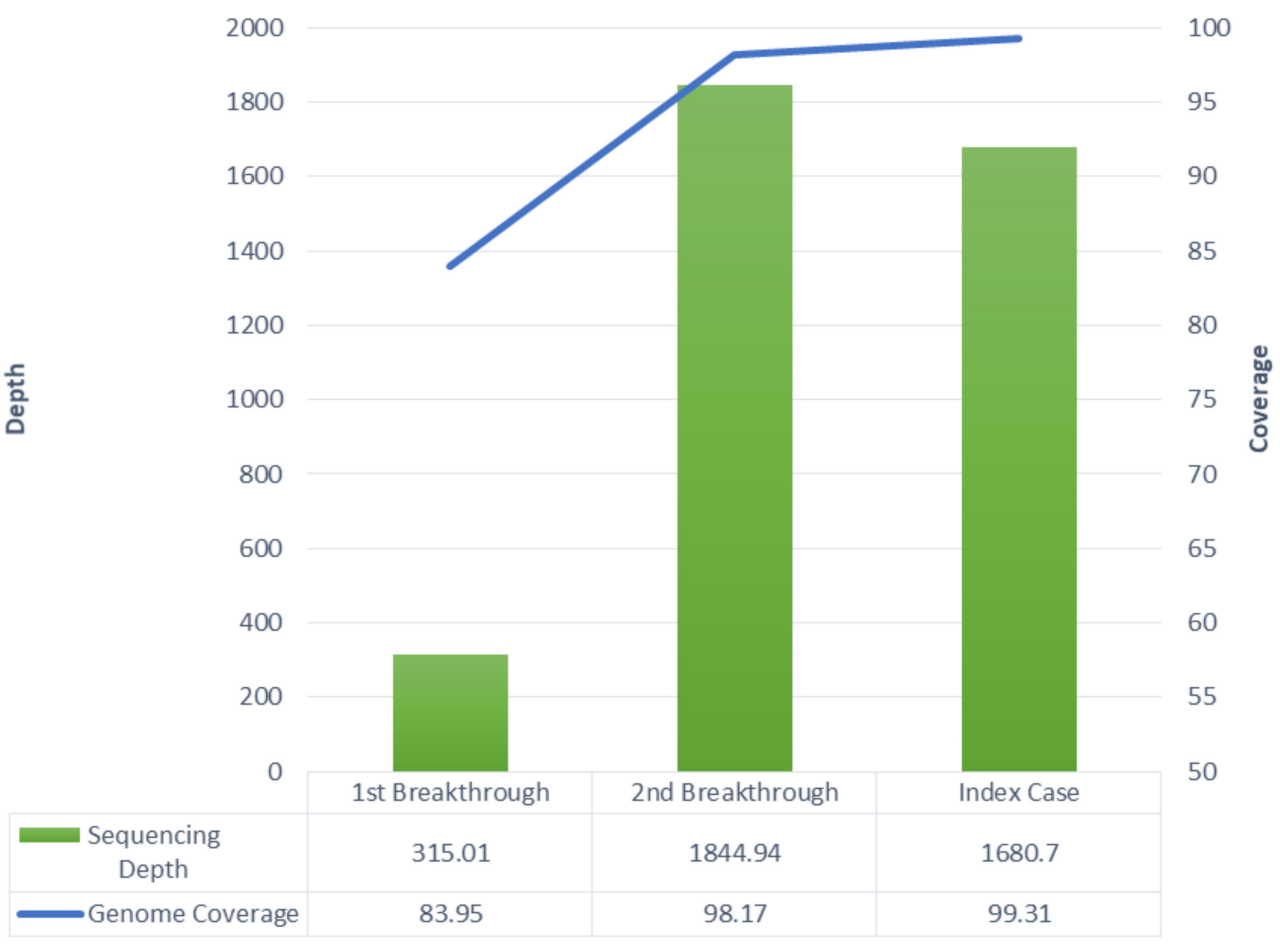
Whole genome sequencing stats including sequencing depth and genome coverage.

The patient's NP + OP samples that were RT-PCR positive for SARS-CoV-2 RNA from 10th April 2021 in the first breakthrough infection and from 3rd May 2021 in the second breakthrough infection were retrieved for sequencing. The presumed index case's NP+OP RT-PCR positive sample from 27th April 2021 was also retrieved for sequencing. All three samples were sequenced together with positive control (SARS-CoV-2 culture RNA) for ascertaining the sequencing efficiency. The sequencing stats are summarized in Figure 2.

The patient's first breakthrough infection was due to the Alpha variant and the second breakthrough infection was due to the Delta variant. The Alpha variant belongs to clade 20I and the Delta variant to clade 21A. The presumed index case's breakthrough infection was also due to the Delta variant.

**Figure 3:**
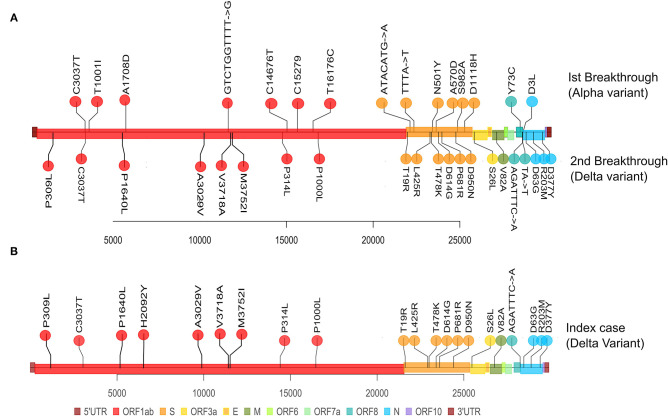
Representation of mutations within SARS-CoV-2 genome for the Patient's paired samples and the single index case sample. The plot displays the different locations of the mutations observed in each sample, with different colors corresponding to different genes. **(A)** Represents the mutations from the Patient's first breakthrough sequence Alpha variant (superiorly) and the second breakthrough sequence Delta variant (inferiorly). **(B)** Represents the mutations from the index case's breakthrough sequence Delta variant (superiorly).

Mutation analysis revealed that the samples from the patient's Delta variant breakthrough infection contained 30 mutations in total: 5 synonymous, 20 non-synonymous, and 4 deletion mutations (Table 3). The presumed index case's samples also contained 30 total mutations: 4 synonymous, 21 non-synonymous mutations, and 4 deletion mutations. Although the second Breakthrough sample and the index case shared the same number of mutations, each differed in one mutation of ORF1a. The second breakthrough sample had a synonymous mutation at ORF1a: G728 (C2449T), which was absent from the index case sample, and the index case showed the presence of a non-synonymous mutation at ORF1a: H2092Y (C6539T), which was not seen in the second breakthrough sample (Table 3). We found 29 shared mutations between the patient and the presumed index case. In particular, the patient and the index case had the same 17 of the 23 mutations that are defining mutation for Delta variant or 21A (Figure 3). This helped us establish that the fully vaccinated family member was the index case from whom the patient acquired the infection.

**Table 3:** List of mutations seen in the three samples.

**Position**	**Gene**	**Amino acid change**	**1st_breakthrough**	**2nd_breakthrough**	**Index case**
C241T	5'UTR	NA			
C1267T	ORF1a	G334G			
C2449T		G728G			
C3037T		F924F			
C3267T		T1001I			
C5184T		P1640L			
C1191T		P309L			
C5388A		A1708D			
C6539T		H2092Y			
C9891T		A3029V			
GTCTGGTTTT11287G		GTCTGGTTTT->G			
T11418C		V3718A			
G11521T		M3752I			
T12946C		Y4227Y			
C12970T		N4235N			
C14408T	ORF1b	P314L			
C14676T		P403P			
C15279T		H604H			
T16176C		T903T			
C16466T		P1000L			
A20262G		L2265L			
C21575T	S	L5F			
C21618G		T19R			
ATACATG21764A		ATACATG->A			
TTTA21990T		TTTA->T			
T22917G		L425R			
C22995A		T478K			
A23063T		N501Y			
C23271A		A570D			
A23403G		D614G			
C23604G		P681R			
G24410A		D950N			
T24506G		S982A			
C24745T		V1061V			
G24914C		D1118H			
C25469T	ORF3	S26L			
T26767C,G	M	V82A			
A28111G	ORF8	Y73C			
AGATTTC28247A		AGATTTC->A			
GAT28280CTA	N	D3L			
A28461G		D63G			
G28881T		R203M			
G29402T		D377Y			
G29427A		R385K			

*The colors highlight the presence of mutation*.

**Figure 4:**
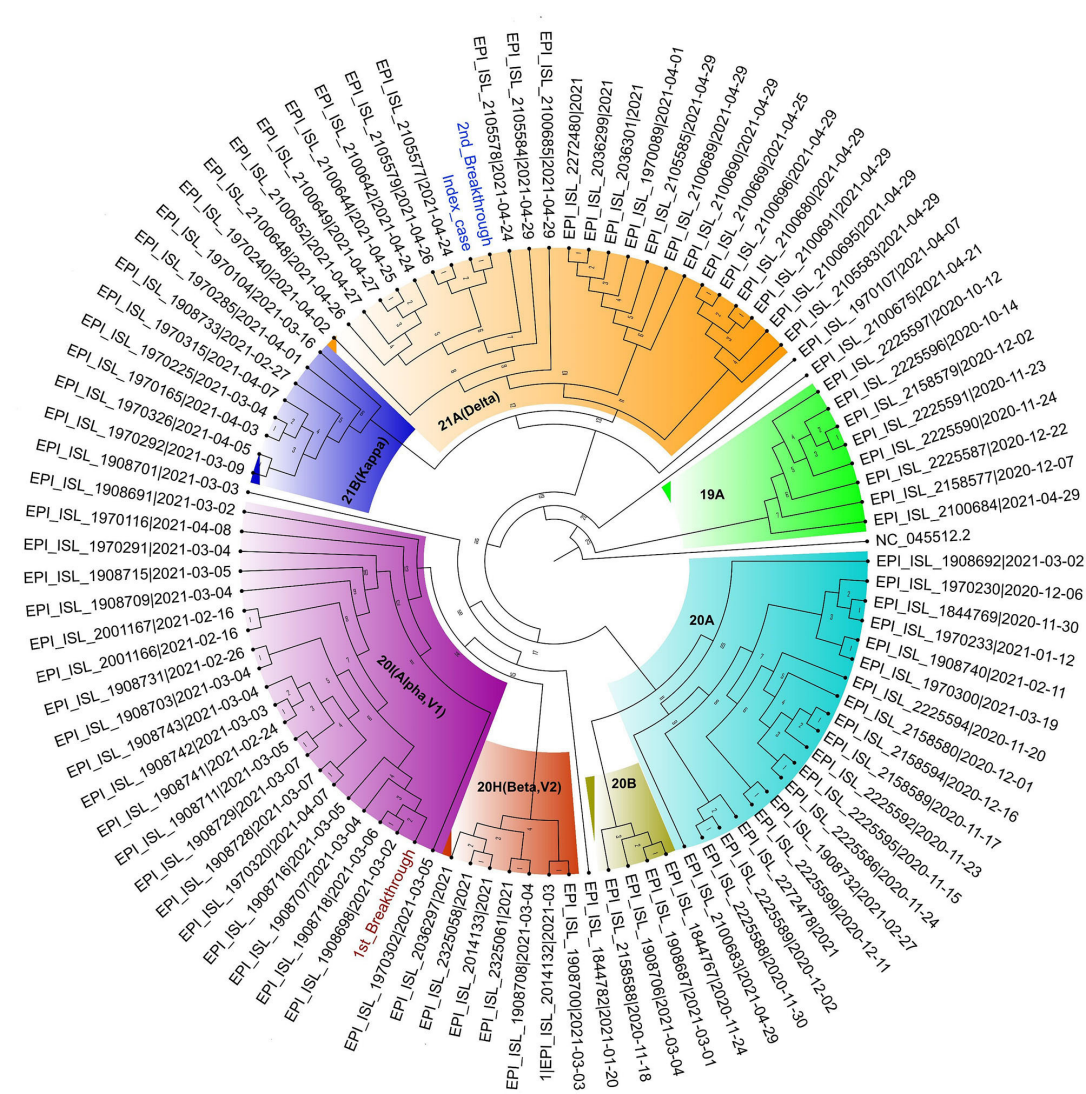
Circular Phylogram of 100 sequences used in the analysis. The Patient's first breakthrough sequence falls in the clade 20I (Alpha, V1) (text in brown) and second breakthrough sequence belongs to clade 21A (Delta) (in blue). The index case sample is also seen to fall close to the second breakthrough sample in clade 21A (Delta) (text in blue).

To evaluate amino-acid alterations, we performed protein-based annotation of all the non-synonymous mutations found, and we evaluated published literature to understand the role of the identified mutations with immune escape potential, as discussed later. The patient's breakthrough infection sequences and the index case's sequence have been represented in a phylogenetic tree in Figure 4.

### Sequence Submission

Both SARS-CoV-2 sequences from the patient and the single sequence from the Index case were submitted to GISAID under the accession numbers EPI_ISL_2811395 (index case), EPI_ISL_2811394 (patient's first breakthrough), EPI_ISL_2811396 (patient's second breakthrough).

## Discussion

Breakthrough infections are defined as infections occurring 2 weeks after complete vaccination (12). While these infections are thought to be rare, it is likely that mild and asymptomatic infections are under-detected. Breakthrough infections are more commonly caused by VOCs (5, 13). Data from the UK suggests that single-dose vaccine efficacy against the Delta variant is reduced (14) and a study in India documented that the Delta variant was over-represented in vaccine breakthrough infections (5). A study looked at over 100 infections in vaccinated health care workers across 3 hospitals in India between March and April 2021. Although breakthrough infections in vaccinated health care workers were not severe, the average size of transmission clusters in these healthcare workers was larger for the Delta Variant (cluster size 3.2) compared to other variants (cluster size 1.1), suggesting that transmission resulting from breakthrough infections due to the Delta variant may be more likely than with other variants (15).

Compared with vaccinated individuals, those who had COVID-19 in the past also have an immune response that offers them some protection. While past infection does confer protection, reinfections may still occur. SARS-CoV-2 reinfections can be proved by whole genome sequencing and confirmed when genetically distinct sequences are available from the two episodes. A probable reinfection is when the virus causing the second infection is identified as a variant in circulation that was not known to be circulating at the time of the first infection. Several studies have documented SARS-CoV-2 reinfections, but reassuringly so far, the risk of reinfection appears to be low (16–18). While most reinfections are thought to be mild (19), a study in India documented reinfections in healthcare workers (20), where the second episode was more severe than the first. Some evidence suggests that those with asymptomatic or mild infections may have less robust and durable protection against future infection (21). A pre-print study estimated that the Delta variant could evade 20–55% of the immune protection provided by prior infection with a non-Delta virus (15), and past infection by itself may not confer sufficient protection from variants of concern in all individuals.

The patient in our study had three distant infections. The first episode was entirely asymptomatic. The RT-PCR positive sample could not be retrieved, and serial COVID-19 serology between this episode and vaccination was negative. Approximately 5–10% of people do not have detectable IgG antibodies following infection, more commonly following asymptomatic infection (22).

The subsequent two infections occurred after complete vaccination and documented seroconversion. They were both symptomatic and additional blood and radiological investigations were available to support the clinical and molecular evidence of COVID-19. In addition, stored samples were available for sequencing. Considering the positive RT-PCR report for the first infection before vaccination and that the first breakthrough infection was caused by the Alpha variant that was not in circulation at the time of the first infection, this may be considered probable reinfection.

The first breakthrough infection was symptomatic and was caused by the Alpha variant. Acute-phase reactant CRP was elevated, and HRCT chest showed COVID-19 pneumonia with CTSS 2/25. As the patient had radiological evidence of lower respiratory tract disease, this was a moderate illness. The patient recovered with treatment which included a course of oral steroids. In this symptomatic episode, the high Ct values were congruent with post-vaccination breakthrough infections that tend to have a lower viral load (23).

During the second breakthrough infection, the patient was infected by Delta variant and developed severe disease requiring hospitalization and supplementary oxygen. Fatigue and cough persisted for a month after discharge and impeded daily activities. Despite vaccination and past infections, the patient experienced a severe and debilitating Delta variant infection. The presence of different variants of concern in the first and the second breakthrough infection confirmed that the Delta variant infection was both a breakthrough infection and confirmed reinfection, an episode we labeled as “breakthrough reinfection.”

Serial serology showed a boosting effect of the humoral immune response after the Alpha variant infection, despite which there was subsequent breakthrough reinfection by the Delta variant. Serology revealed boosting after the Delta variant infection as well.

The time interval between the two breakthrough infections was short, with just 4 days between symptom resolution in the first and symptom onset in the second. However, several different lines of evidence show that the second and third episodes were distinct infections caused by different variants. Samples from the two episodes show two different variants that fit the criteria for best evidence for reinfection (24). However, the time period between the positive tests in the two episodes was just 21 days. In addition to genomic evidence, there was evidence from clinical history, negative RT-PCR tests, acute phase reactants, and chest imaging that support resolution of the first infection before a new infection occurred. Clinically the patient's symptoms had completely resolved for 4 days before symptoms reappeared in the third episode. There were two negative RT-PCR results between the second and third episode. CRP had normalized between the episodes. Chest imaging (HRCT) showed resolution of pulmonary involvement between the two episodes with new and progressive changes during Delta variant infection.

The short time interval between the two episodes of breakthrough infection highlights that the existing criteria for reinfection that use predefined periods may result in missing reinfections by the Delta variant. Following time-based criteria, such as those in the US CDC investigation protocol for reinfections (24), would have resulted in missing this important case of breakthrough reinfection. With the emergence of VOCs, clinicians need to be alert to the possibility of reinfections occurring in close succession. It would be prudent to use clinical, molecular, biochemical, serological, and radiological clues to assign reinfections.

It is epidemiologically relevant that we established that the patient acquired Delta variant infection from a fully vaccinated family member. The index case and the patient lived together, and the index case developed symptoms 3 days before the patient. The patient was still self-isolating at home while recovering from the Alpha variant infection, and there was no other potential exposure at home. Whole-genome sequencing of samples from the index case and the patient both identified Delta variant and mutation analysis identified 29 shared mutations between them. There was only one unique mutation between their samples, H2092Y in the ORF1ab region of SARS-CoV-2. High sequencing depth and genome coverage of the samples give credence to the findings and insights therein. Incidentally, this was also the time when India was in the middle of the Delta variant driven second wave.

During the patient's breakthrough reinfection with the Delta variant, Ct values were as low as 18.20 on confirmatory genes. A study on nosocomial transmission of SARS-CoV-2 between patients sharing a hospital room found that Ct values 21 or lower were associated with transmission (25). Despite vaccination, the patient in our study had Ct values low enough to suggest transmission potential. The patient herself acquired the infection from a fully vaccinated close contact. As Delta variant infections pose a threat of high transmissibility, precautions like wearing a mask will continue to be relevant to protect both vaccinated individuals, immunocompromised individuals, and naive individuals.

The first breakthrough infections occurred over three and a half weeks after the second dose of COVISHIELD and documented seroconversion with anti-RBD IgG antibodies. Serology on March 21st, 6 days after the second dose, showed a CLIA index of 5.84. Repeated serology on April 7th, 3 days before symptoms onset in the first breakthrough episode, showed a decreased CLIA index of 2.85. It is possible that the decrease in antibodies may have contributed to increased susceptibility to Alpha variant infection. After the first breakthrough infection by the Alpha variant, there was the boosting effect seen on serology. Breakthrough reinfection with Delta variant occurred despite this. There may be higher humoral correlates of protection for some VOCs.

COVID-19 serology was performed using different methodologies and platforms, with some of the assays targeting antibodies against different antigens. As we were unable to retrieve the samples, we could not repeat serological tests using a harmonized protocol and reported in the same units (BAU/ml). Fortunately, a few serial serological tests were conducted at the same lab using the same methodology and same assays, allowing for comparison. As neutralizing antibodies weren't measured, it is possible that the patient had anti-Spike and anti-RBD IgG without having sufficient neutralizing antibodies to prevent breakthrough reinfection. Cellular immunity was not assessed. It is possible the patient had a poor CD8+ T cellar response, which resulted in severe disease.

It is possible that steroids prescribed during the first breakthrough infection contributed to susceptibly to reinfection by Delta variant. The use of steroids during COVID-19 may delay the development of immunity following infection, and such individuals may be more susceptible to early reinfection by a VOC. This may be relevant to patients prescribed steroids and housed together in common COVID-19 wards, especially in the context of different VOCs in circulation.

Some VOCs like the Beta variant and Delta variant have been characterized to partially evade post-vaccination immunity. Despite the presence of numerous receptor-binding domain (RBD) mutations, strains such as B.1.1.7 remain potently neutralized by vaccine-elected sera. Several anti-RBD-specific antibodies can only bind to the open spike protein (26); it is possible that mutations altering the conformation of the spike protein may make the RBD less susceptible to neutralizing antibodies by decreasing the chance of the open conformation (27). Some mutations, combined with others, could make the virus more infectious. For example, Leucine-452 is found in the RBD receptor-binding motif, where it interacts directly with the ACE2 receptor. Its replacement with arginine is predicted to result in higher receptor affinity and resistance to neutralizing antibodies (28).

The structural analysis of RBD mutations, L452R and E484Q, and P681R in the furin cleavage area, highlight the likelihood of increased ACE2 binding and S1-S2 cleavage rate, resulting in better transmissibility. The identical two RBD mutations result in decreased binding to selected monoclonal antibodies (mAbs) and lowering mAb neutralizing activity (29). The ORF1ab mutation, A1708D, may reduce CD8+ T cell activation and thereby play a role in immune evasion (30). L5F mutation is seen to increase the epitope binding affinity for 37 different HLA alleles (31, 32). The TTTA->T deletion in Spike protein is predicted to change the structure of the N3 NTD loop (amino acid positions 140–156) and has been shown to impact neutralization by a variety of neutralizing antibodies (33). T478K affects the Spike binding domain with the human receptor ACE2, raising the electrostatic potential at the interface and may represent a genetic pathway for SARS-CoV-2 to avoid immune detection (34). N501Y is one of six essential amino acids involved in the development of the RBD-hACE2 complex. As a result, this mutation may allow a greater interaction between the spike protein and hACE2 (35). Thus genetic alterations may impair the host immune system's capacity to recognize and resist the virus (36, 37). Certain mutations may enhance viral spread from cell to cell by fusion. P681R mutation may enhance cell fusion and help the virus evade neutralizing antibodies (38, 39).

Although limited to a single patient, our study reiterates that rare cases of severe breakthrough infection and reinfection can occur with VOCs at a short time interval. These infections can have Ct values low enough to pose transmission risk, and breakthrough infections with the Delta variant can result in secondary transmission.

We are mindful that some may misinterpret our work to mean that widespread severe breakthrough infection and reinfections are likely; however, we would like to clearly state that our study is based on one patient, and no such conclusion can be made. The patient survived infection by two VOCs, and it is very likely that vaccination provided some protection.

## Conclusion

Our study identifies a rare breakthrough infection that was also a confirmed reinfection. We propose the term “breakthrough reinfection” for such episodes. In this study, the patient had breakthrough reinfection severe enough to result in hypoxia and hospitalization. The patient was infected by a fully vaccinated family member, and the patient's Ct values in the Delta variant breakthrough reinfection were low enough to suggest transmission potential. This reinforces that fully vaccinated individuals should also continue to take precautions to protect themselves and others. The Alpha variant breakthrough infection occurred despite documented seroconversion, and the Delta variant breakthrough reinfection occurred despite boosting by prior infection. Seroconversion by itself may not be enough to prevent infection. With VOCs, there may be a short time interval between infections, and current time criteria to suspect reinfection may need to be revised in view of VOCs. There is an urgent need to understand the patient-specific variables such as pre-existing illness or immunological factors, including deficient humoral and cellular immune responses, that predispose to breakthrough reinfections.

## Data Availability Statement

Both SARS-CoV-2 sequences from the patient and the single sequence from the Index case were submitted to GISAID under the accession numbers EPI_ISL_2811395 (index case), EPI_ISL_2811394 (patient's first breakthrough), EPI_ISL_2811396 (patient's second breakthrough).

## Ethics Statement

The studies involving human participants were reviewed and approved by the Institutional Review Board of Kasturba Hospital of Infectious Diseases; IRB number 015/2020. The patients/participants provided their written informed consent to participate in this study. Written informed consent was obtained from the individual(s) for the publication of any potentially identifiable images or data included in this article.

## Author Contributions

JS and SP conceptualized the study. SP, VA, SA, and NC collected and compiled data from different sources. PD, PM, and RP performed genome sequencing and analyses. SP, JS, VA, and RP drafted the manuscript. SP, PD, PM, and RP prepared tables and figures. RS evaluated chest imaging. JS, RP, and VA provided resources. All authors contributed to data interpretation, contributed to the writing, critically reviewing, revising, and approving the final manuscript for submission.

## Conflict of Interest

The authors declare that the research was conducted in the absence of any commercial or financial relationships that could be construed as a potential conflict of interest.

## Publisher's Note

All claims expressed in this article are solely those of the authors and do not necessarily represent those of their affiliated organizations, or those of the publisher, the editors and the reviewers. Any product that may be evaluated in this article, or claim that may be made by its manufacturer, is not guaranteed or endorsed by the publisher.
