# Supplementary material for: Severe SARS-CoV-2 Breakthrough Reinfection With Delta Variant After Recovery From Breakthrough Infection by Alpha Variant in a Fully Vaccinated Health Worker
Source: Front Med (Lausanne). 2021 Aug 20;8:737007. doi: 10.3389/fmed.2021.737007 (PMC8418387; doi:10.3389/fmed.2021.737007)
Supplement: Supplementary file 1 [file Table_1.docx]

**Supplementary Material**

| Supplementary Table 1: RT-PCR Methodolgy | | | |
| --- | --- | --- | --- |
| Infection episode and Vaccination | RT-PCR Date and Result | RT-PCR Methodology | Molecular Lab |
| First Episode | 16/08/2020 Positive | NP+OP swabs, TRUPCR kit on Quant Studio 5 by Thermo Fisher, Gene targets | Lab 1 |
|  | 19/08/2020  Negative | NP+OP swabs, TRUPCR kit on CFX 96 dx-Bio-Rad | Lab 2 |
| Second Episode (First breakthrough infection) | 12/04/2021 Positive | NP+OP swabs, TRUPCR kit on CFX 96 dx-Bio-Rad | Lab 2 |
|  | 14/04/2021 Negative | NP+OP swabs, TRUPCR kit on CFX 96 dx-Bio-Rad | Lab 2 |
|  | 24/04/2021 Negative | NP+OP swabs, TRUPCR kit on CFX 96 dx-Bio-Rad | Lab 2 |
| Third Episode (Second breakthrough infection, First proven reinfection) | 03/05/2020 Positive | NP+OP swabs, TRUPCR kit on CFX 96 dx-Bio-Rad | Lab 2 |
|  | 04/05/2021 Positive | NP+OP swabs, TRUPCR kit on CFX 96 dx-Bio-Rad | Lab 2 |
|  | 08/05/2021  Positive | NP+OP swabs, TRUPCR kit on CFX 96 dx-Bio-Rad | Lab 2 |
|  | 15/05/2021  Negative C | NP+OP swabs, details unavailable | Lab 3 |
|  | 18/05/2021  Negative | NP+OP swabs, TRUPCR kit on CFX 96 dx-Bio-Rad | Lab 2 |

Key:

NP + OP - Nasopharyngeal plus oropharyngeal

Lab - Laboratory
